# The role of Rubisco kinetics and pyrenoid morphology in shaping the CCM of haptophyte microalgae

**DOI:** 10.1093/jxb/erx179

**Published:** 2017-06-03

**Authors:** Ana M C Heureux, Jodi N Young, Spencer M Whitney, Maeve R Eason-Hubbard, Renee B Y Lee, Robert E Sharwood, Rosalind E M Rickaby

**Affiliations:** 1 University of Oxford, Department of Earth Sciences, South Parks Road, Oxford, UK; 2 University of Washington, School of Oceanography, Seattle, WA, USA; 3 ARC Centre of Excellence for Translational Photosynthesis, Research School of Biology, Australian National University, Canberra ACT, Australia; 4 University of Reading, School of Biological Sciences, Reading, Berkshire, UK

**Keywords:** Algae, carbon-concentrating mechanisms, Haptophyta, pyrenoid, Rubisco

## Abstract

The haptophyte algae are a cosmopolitan group of primary producers that contribute significantly to the marine carbon cycle and play a major role in paleo-climate studies. Despite their global importance, little is known about carbon assimilation in haptophytes, in particular the kinetics of their Form 1D CO_2_-fixing enzyme, Rubisco. Here we examine Rubisco properties of three haptophytes with a range of pyrenoid morphologies (*Pleurochrysis carterae*, *Tisochrysis lutea*, and *Pavlova lutheri*) and the diatom *Phaeodactylum tricornutum* that exhibit contrasting sensitivities to the trade-offs between substrate affinity (*K*_m_) and turnover rate (*k*_cat_) for both CO_2_ and O_2_. The pyrenoid-containing *T. lutea* and *P. carterae* showed lower Rubisco content and carboxylation properties (*K*_C_ and *k*^C^_cat_) comparable with those of Form 1D-containing non-green algae. In contrast, the pyrenoid-lacking *P. lutheri* produced Rubisco in 3-fold higher amounts, and displayed a Form 1B Rubisco *k*^C^_cat_–*K*_C_ relationship and increased CO_2_/O_2_ specificity that, when modeled in the context of a C_3_ leaf, supported equivalent rates of photosynthesis to higher plant Rubisco. Correlation between the differing Rubisco properties and the occurrence and localization of pyrenoids with differing intracellular CO_2_:O_2_ microenvironments has probably influenced the divergent evolution of Form 1B and 1D Rubisco kinetics.

## Teaser

Variations in the pyrenoid morphology, Rubisco content, and kinetics among haptophyte algae provide insight into their carbon-concentrating mechanisms.

## Introduction

**Fig. 1.**
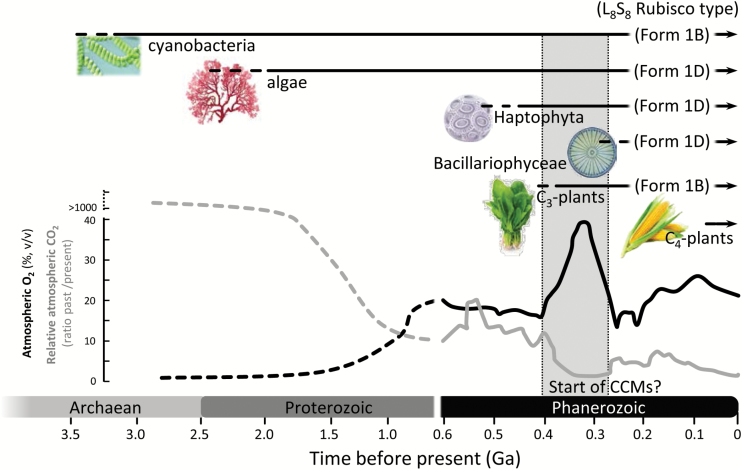
Rubisco evolution and catalysis. Geological history of the past versus present atmospheric [CO_2_] (gray) and percentage atmospheric O_2_ (% v/v) (black; modified from Berner and Canfield, 1989; Badger *et al.*, 2002; Whitney *et al.*, 2011) highlighting the estimated appearance of key primary producers (horizontal lines) (Yoon *et al.*, 2002, 2004; Liu *et al.*, 2010) their differing Form 1B or ID Rubisco lineages they produce, and the predicted timing when algal carbon-concentrating mechanisms (CCMs; gray shading) evolved (Badger *et al.*, 2002; Moritz and Griffiths, 2013).

The CO_2_-fixing enzyme Rubisco (EC 4.1.1.39) evolved in the Archaean Eon when the atmosphere lacked O_2_, and CO_2_ was estimated to be 50-fold higher than current levels (Berner and Canfield, 1989; Berner, 2006; Tabita *et al.*, 2008). With the evolution of O_2_-producing photosynthesis around the early Proterozoic (2.5 Gya), atmospheric O_2_ increased while the CO_2_ concentration declined (Canfield, 2005) (Fig. 1). The diminishing atmospheric CO_2_:O_2_ ratio negatively influenced Rubisco catalysis, as its photosynthetic CO_2_-fixing function is competitively inhibited by O_2_ to produce 2-phosphoglycolate (2-PG) (Tcherkez *et al.*, 2006; Whitney *et al.*, 2011; Sharwood, 2017). Recycling of 2-PG to 3-phosphoglycerate (3-PGA) by photorespiration consumes energy and loses fixed CO_2_. A further limitation to Rubisco function in the modern atmosphere is a low affinity for CO_2_ and a slow catalytic rate that necessitates high Rubisco concentrations to support adequate rates of photosynthesis (Reinfelder, 2011; Whitney *et al.*, 2011; Long *et al.*, 2015; Sharwood *et al.*, 2016*b*).

The catalytic limitations of Rubisco are exacerbated in aquatic ecosystems due to restraints on aqueous CO_2_ availability because of slow rates of gas diffusion in water (~10000 times slower than in air) and reliance on mixing of the water column (Badger *et al.*, 1998). In addition, increased partitioning of inorganic carbon to HCO_3_^–^ with higher pH in aquatic systems diminishes aqueous CO_2_ availability. There is evidence of catalytic adaptation by Rubisco in algae to the changing atmospheric CO_2_ (and O_2_) conditions over geological time scales (Young *et al.*, 2012). Recent work, however, showed that the characteristic faster CO_2_ fixation rates (*k*^C^_cat_) and lower CO_2_ affinities (i.e. higher *K*_m_ for CO_2_; *K*_C_) observed in Form 1A and Form 1B Rubisco [e.g. in *Chlamydomonas* with a pyrenoid-based CO_2_-concentrating mechanism (CCM)] (Badger *et al.*, 1998; Ghannoum *et al.*, 2005; Sharwood *et al.*, 2016*a*) are not shared by diatom Form 1D Rubisco (Hanson, 2016; Young *et al.*, 2016; see also Fig. 3A). This has led to calls for a more expansive analysis of Rubisco’s natural kinetic diversity so that we can fully understand the correlative interactions between specificity for CO_2_ as opposed to O_2_ (*S*_C/O_), *k*^C^_cat_, and *K*_C_. The one-dimensional, linear correlations previously proposed (Tcherkez *et al.*, 2006; Savir *et al.*, 2010) may actually vary with photosynthetic taxa (Tcherkez, 2013, 2016; Hanson, 2016; Sharwood, 2017).

In photosynthetic organisms, the CCM arose multiple times in response to a declining atmospheric CO_2_:O_2_ ratio as a means to increase the CO_2_/O_2_ environment around Rubisco (Fig. 1). Data on the anatomical, biochemical, and genomic detail for CCMs in vascular plants with C_4_ and Crassulacean acid metabolism (CAM) physiologies are highly detailed (von Caemmerer and Furbank, 2016). The high CO_2_ environment reduces Rubisco oxygenation and the associated energy costs of photorespiration, allowing the plant to work with lower stomatal conductance and reduced amounts of Rubisco (Sage *et al.*, 2012). These features allow more efficient use of water, nitrogen, and light, and permit these plants to survive in more arid and nutrient-limited environments (Sage, 2002; Ghannoum *et al.*, 2005; Lara and Andreo, 2011; Long *et al.*, 2015). The CCM in plants also allowed Form 1B Rubisco to evolve a higher *k*^C^_cat_ at the expense of a higher *K*_C_ (i.e. lower CO_2_ affinity) with little or no effect on *S*_C/O_ (Sharwood *et al.*, 2016a, b). Curiously this *k*^C^_cat_–*K*_C_ trade-off is not shared by Form 1D Rubisco from diatoms where relatively higher *K*_C_ values have been retained as a consequence of other environmental pressures (low nutrient and extracellular CO_2_ availability) that pose limitations to resource investment into Rubisco (Young *et al.*, 2016). It is likely that resources other than CO_2_, such as nitrogen and light availability, have a strong influence on CCM evolution and regulation (Raven *et al.*, 2008, 2012).

Understanding how microalgal Rubisco catalysis has differentially evolved remains limited by our understanding of the structural components and effectiveness of the CCM in microalgae. The last few years have seen significant advances in our understanding of CCM in the model freshwater green alga, *Chlamydomonas reinhardtii* (Engel *et al.*, 2015; Wang *et al.*, 2015; Yamano *et al.*, 2015; Mackinder *et al.*, 2016; Mangan *et al.*, 2016; Wang *et al.*, 2016). To what extent this knowledge is translatable to the CCM of the structurally differing and evolutionarily distinct marine microalgae (e.g. diatoms and haptophytes) remains unclear (Bedoshvili *et al.*, 2009; Hopkinson *et al.*, 2011, 2013). Currently a completed nuclear haptophyte genome is available for the Isochrysidale *Emiliania huxleyi* (Read *et al.*, 2013) and a draft genome for the Prymnesiale *Chrysochromulina tobin* (Hovde *et al.*, 2015). Although less understood, the CCMs of marine microalgae are known typically to employ a pyrenoid, Rubisco activase, carbonic anhydrase (CA), and inorganic carbon (C_i_) transporters to elevate CO_2_ levels around Rubisco (Hopkinson *et al.*, 2011; Reinfelder, 2011; Loganathan *et al.*, 2016). The pyrenoid is a proteinaceous body that appears electron dense when examined by TEM and contains most, sometimes all, of the cellular Rubisco (Engel *et al.*, 2015; Mackinder *et al.*, 2016).

In *C. reinhardtii*, HCO_3_^–^ transport occurs via thylakoids and C_i_ transporters that work in association with pyrenoid CAs to elevate CO_2_ around Rubisco (Karlsson *et al.*, 1998; Trimborn *et al.*, 2007; Lee *et al.*, 2013; Wang *et al.*, 2015; Yamano *et al.*, 2015). Physiological and genetic evidence in model diatoms imply that HCO_3_^–^ is pumped into the chloroplast stroma and diffuses into the pyrenoid where it is converted to CO_2_ by CA to elevate the [CO_2_] around Rubisco (Hopkinson *et al.*, 2011, 2016). Recent identification of a thylakoid lumen-localized CA in *P. tricornutum* further suggests that the pyrenoid-penetrating thylakoids probably provide an important CO_2_ supply within the pyrenoids (Kikutani *et al.*, 2016).

**Fig. 2.**
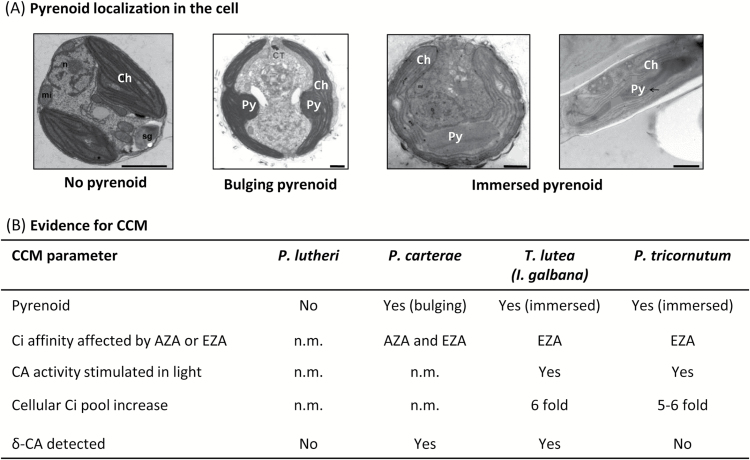
Microalgae pyrenoid and CCM composition. (A) TEM images were compiled from the literature to represent the range of pyrenoids presented in this. To represent a pyrenoid lacking Pavlovale, we use *Pavlova viridis* from Bendif *et al.* (2011) (Protist, 162, Bendif EM, Probert I, Hervé A, Billard C, Goux D, Lelong C, Cadoret JP, Véron B. Integrative taxonomy of the Pavlovophyceae (Haptophyta): a reassessment, 738–761, ©2011, with permission from Elsevier) as the TEM image clearly represents the lack of pyrenoid. *Pavlova lutheri* is visualized in the same study; however, the TEM image does not show the chloroplast (Ch) lacking a pyrenoid as clearly. TEM image of *P. carterae* from Beech and Wetherbee (1988) [republished with permission of the International Phycological Society from Observations on the flagellar apparatus and peripheral endoplasmic reticulum of the coccolithophorid, Pleurochrysis carterae (Prymnesiophyceae), Beech PL, Wetherbee R, Phycologia 27, 1988; permission conveyed through Copyright Clearance Center, Inc.] illustrates pyrenoids (Py) bulging toward the center of the cell, and the two species *T. lutea* (Bendif *et al.*, 2014) (Journal of Applied Phycology, Erratum to: On the description of Tisochrysis lutea gen. nov. sp. nov. and Isochrysis nuda sp. nov. in the Isochrysidales, and the transfer of Dicrateria to the Prymnesiales (Haptophyta), 26, 2014, 1617, Bendif EM, Probert I, Schroeder DC, de Vargas C. With permission of Springer) and *P. tricornutum* (Allen *et al.*, 2011) (Allen AE, Moustafa A, Montsant A, Eckert A, Kroth PG, Bowler C. Evolution and functional diversification of fructose bisphosphate aldolase genes in photosynthetic marine diatoms. Molecular Biology and Evolution 2012, 29, 367–279, by permission of Oxford University Press) show pyrenoids immersed within the chloroplast. (B) Summary of published experimental evidence for the presence of a CCM in the species with a pyrenoid. Evidence for a CCM is detectable by: (i) inhibition of CO_2_ assimilation by the impermeable acetazolamide (AZA) or membrane-permeable ethoxyzolamide (EZA) CA inhibitors (Burns and Beardall, 1987; Okazaki *et al.*, 1992; Badger *et al.*, 1998; Hopkinson *et al.*, 2013); (ii) stimulation of CA activity following cell illumination (Badger *et al.*, 1998); (iii) whether the intercellular C_i_ pool is higher than the external environment (Badger *et al.*, 1998); or (iv) the preliminary detection of δ-CA using methods described in the Materials and methods.

What remains unclear is how the pyrenoid structure influences CCM efficiency. In *C. reinhardtii*, the pyrenoid contains a starch sheath (Moroney *et al.*, 2011; Engel *et al.*, 2015) while in diatoms it can comprise a lipid membrane (Bedoshvili *et al.*, 2009), lack a delimiting structure (Bendif *et al.*, 2011), vary in number and shape, and differ in the presence/structure of traversing thylakoids (Badger *et al.*, 1998). To better understand the relationships between Rubisco kinetics, content, and pyrenoid biology in marine microalgae, we have expanded on our previous study of diatom Rubisco (Young *et al.*, 2016) to include three marine haptophytes that contain bulging (*Pleurochrysis carterae*), immersed [*Tisochrysis lutea*, formerly *Isocrysis* sp. strain CS-177 (Bendif *et al.*, 2013)], or no pyrenoid (*Pavlova lutheri*) within their chloroplast and varying numbers and location of pyrenoid-traversing thylakoids (Fig. 2A).

## Materials and methods

### Algae culturing

Cultures of the haptophytes *P. lutheri* (CS-182), *P. carterae* (CS-287), *T. lutea* [CS-177; original strain name *Isochrysis* sp. (Bendif *et al.*, 2013)], and the diatom, *Phaeodactylum tricornutum* (CS-29) were obtained from the Australian National Algae Culture Collection CSIRO (https://www.csiro.au/en/Research/Collections/ANACC) and grown at 20 °C in 0.2 μm filtered and autoclaved seawater containing f/2 (Guillard and Ryther, 1962) or GSe (*P. carterae*; Blackburn *et al.*, 2001) nutrients, vitamins, and trace metals. The cultures were grown in polycarbonate culture flasks under 150 ± 50 μmol photons m^–2^ s^–1^ illumination on a 16:8 h light:dark cycle.

### Pyrenoid morphology and CCM characterization.

Details of pyrenoid morphology and estimates of C_i_ and CA pools for the microalgae were compiled from the literature (Manton and Peterfi, 1969; Billard and Gayral, 1972; Green, 1975; Green and Pienaar, 1977; Borowitzka and Volcani, 1978; Hori and Green, 1985; Badger *et al.*, 1998; Bendif *et al.*, 2011, 2013). A preliminary screen for putative δ-CA genes was carried out using PCR. δ-CA is a functional carbonic anhydrase, as demonstrated *in vitro* by Del Prete *et al.* (2014) and Lee *et al.* (2013). It is regulated by CO_2_, and is thus important in inorganic carbon acquisition (Lane and Morel, 2000*a*).

Genomic DNA was extracted as described (Richlen and Barber, 2005) and candidate δ-CA genes were amplified by PCR using primers Δfwd (5'-GTTGGCGAGACGTACGAGGTGCACTGG-3') and Δrev (5'- GCGATCGACCTGCCAGGTGATGGG-3') that were designed to the conserved C-terminal amino acid sequences VGETYEVHW and PITWQVDR, respectively. The ~370 bp DNA product amplified from *P. carterae* and *Isochrysis galbana* (for comparison with *T. lutea*) was sequenced by Source BioScience (Oxford, UK). Confirmation that the absence of δ-CA from our *P. tricornutum* (strain CCAP1055/1 a monoclonal culture derived from a fusiform cell in May 2003 from strain CCMP632) was further supported by analysis for δ-CA homologs within the fully sequenced genome of *P. tricornutum* (Bowler *et al.*, 2008) and its predicted protein products. Similarly, the absence of δ-CA was further confirmed by a search of the Pavlovales sp. CCMP2436 (JGI) genome sequence.

A BLAST search of the genomes was carried out using the δ-CA protein sequence from *Thalassiosira pseudonana* (BAO52718) and *Thalassiosira weissflogii* (AAV39532), both centric diatoms, and *Fragilariopsis cylindrus* CCMP1102 (OEU11320), a pennate diatom, as query sequences. The BLAST search yielded no hits. Together with our PCR, we concluded that there were no δ-CA homologs in this strain of *P. tricornutum*. A BLAST search was also carried out on the genome of Pavlovales sp. CCMP2436 (JGI), an environmental isolate using the δ-CA protein sequence from *T. pseudonana* (BA052718) and the haptophytes *Emiliania huxleyi* (ABG37687), *I. galbana* (EC146202, EC142695), and *Chrysochromulina* sp. CCMP291 (KO021563, KO028292). Although this genome is not fully curated and the culture has not been taxonomically described, the preliminary search yielded no hits.

### Rubisco extraction and kinetic assessment

Algal cells were harvested via centrifugation (2000 *g* for 10 min) and the pelleted cells snap-frozen in liquid nitrogen and stored at –80 °C until assay. The crude soluble cell extracts were obtained by rupturing cells using a French press as described previously (Young *et al.*, 2016). As detailed in the same study, Rubisco content was quantified by [^14^C]CABP (2-carboxyarabinitol 1,5-bisphosphate) binding within the crude extract and concentrations of soluble protein were quantified using the Bradford assay against BSA. Rubisco catalytic parameters: maximum carboxylation rate (*k*^C^_cat_) and half-saturation constants for CO_2_ and O_2_ (*K*_C_ and *K*_O_, respectively) were measured at 25 °C using ^14^CO_2_ fixation assays employing crude extract that had been activated for 10–15 min at 25 °C with 10 mM MgCl_2_ and 10 mM NaHCO_3_. The CO_2_ concentrations in the ^14^CO_2_ assays were calculated using the Henderson–Hasselbalch equation and the parameters detailed in (Sharwood *et al.*, 2016*a*). Measurements of *S*_C/O_ were made using Rubisco rapidly purified from ~1 g of pelleted algal cells as described (Young *et al.*, 2016).

### Simulating the influence of microalgae Rubisco on C_3_ plant photosynthesis

The carboxylase activity-limited assimilation rates were simulated according to Farquhar *et al.* (1980) using the equation:

A=(Cc.sc−0.5scoo_)kcatc.BCc.sc+K(1+oKo)−Rd

assuming a CO_2_ solubility in H_2_O (*s*_c_) of 0.0334M bar^–1^, an *O* of 267 μM, a Rubisco content (*B*) of 20 µmol catalytic sites m^–2^, and a non-photorespiratory CO_2_ assimilation rate (*R*_d_) of 2 µmol m^–2^ s^–1^. Under higher chloroplast CO_2_ pressures (*C*_c_), the photosynthetic rate becomes light- (or electron transport rate, ETR-) limited and is modeled according to the equation:

$$\text{A}=\frac{\left(C_{c}{\text{.s}}_{\text{c}}-0.5_{s_{\frac{c}{o}}}^{\underline{o}}\right)}{4\left(C_{c}\text{.}s_{c}{+}_{s_{\frac{c}{o}}}^{\underline{o}}\right)}-R_{\text{d}}$$

assuming an electron transport rate (*J*) of 150 µmol m^–2^ s^–1^.

## Results and Discussion

### The differing pyrenoid morphologies within the microalgae studied

A central objective of this study was to examine the correlations between pyrenoid morphology, evidence of a CCM, and the content and catalysis of Rubisco in microalgae. As summarized in Fig. 2A, *T. lutea* possesses a pyrenoid immersed in the center of the plastid with 1–2 thylakoids traversing the center of the pyrenoid (Bendif *et al.*, 2013; Borowitzka and Volcani, 1978). In contrast, the pyrenoid in *P. carterae* bulges out from the plastid toward the center of the cell, with 5–6 continuous thylakoids traversing the plastid and pyrenoid (Manton and Peterfi, 1969; Beech and Wetherbee, 1988). Immersed versus bulging pyrenoids differ in the location within the cell, relative separation from the plastid (i.e. the presence of a lipid membrane has been suggested from TEM observations), and the connectivity to plastid thylakoids. A lipid membrane has been observed around the immersed/semi-immersed pyrenoids of two other members of the Isochrysidales—*Chrysotila lamellosa* (Billard and Gayral, 1972; Green and Parke, 1975) and *Isochrysis galbana* (Green and Pienaar, 1977). However, Bendif *et al.* (2013) did not detect a membrane around the pyrenoid of *T. lutea* nor has one been observed around pyrenoids of the bulging morphotype in any haptophyte species, including *P. carterae*. In the Pavlovophyceae, the pyrenoids are often bulging towards the exterior of the cell—albeit not in *P. lutheri* where no pyrenoid is apparent (Green, 1975; Burris, 1981; Bendif *et al.*, 2011). *Phaeodactylum tricornutum* was included in this study and, like many members of the lineage, has pyrenoids that are fully immersed within the chloroplast with 1–2 pyrenoid-traversing thylakoids (Borowitzka and Volcani, 1978; Bedoshvili *et al.*, 2009) (Fig. 2A).

### Experimental evidence for a CCM

A key component of a CCM is the enzyme CA that catalyzes the rapid interconversion between CO_2_ and HCO_3_^–^. In marine primary producers, the CA activity of a CCM is particularly beneficial for accessing CO_2_ from the high oceanic HCO_3_^–^ concentrations. The effects of two CA inhibitors, the membrane-permeable ethoxyzolamide (EZA) and the relatively impermeable acetazolamide (AZA), are commonly used to test for CCM activity (Okazaki *et al.*, 1992). This is undertaken by examining the influence of EZA and AZA on the affinity of photosynthesis for plasma membrane-based inorganic carbon (C_i_) (Okazaki *et al.*, 1992; Badger *et al.*, 1998) and light-stimulated CA activity (Burns and Beardall, 1987).

Using modern cell biology tools, there have been significant advances in understanding the CCM components in microalgae (Engel, 2015) which includes the discovery of novel CA isoforms and their intercellular localization (Jin *et al.*, 2016; Kikutani *et al.*, 2016). While a comparable detailed analysis of haptophyte CCM components is beyond the scope of this work, Fig. 2B summarizes the known CCM features in the microalgae studied here. EZA treatment reduces the affinity for C_i_ in photosynthesis for the pyrenoid-containing *I. galbana* (as a proxy for *T. lutea*), *P. tricornutum*, and *P. carterae* species, with the external CA inhibitor AZA also affecting the C_i_ affinity in *P. carterae* (Badger *et al.*, 1998; Chen *et al.*, 2006; Hopkinson *et al.*, 2013). We note that other diatoms with fully immersed pyrenoids have been shown to be sensitive to AZA (Hopkinson *et al.*, 2013). The influence of EZA or AZA on the photosynthetic carbon assimilation rate in *P. lutheri*, the species lacking a pyrenoid, remains untested. Similarly, the light-stimulated CA activity found in *P. tricornutum* and *T. lutea* has yet to be examined in *P. carterae* and *P. lutheri* (Fig. 2B). It is estimated that the CCMs associated with immersed pyrenoids can increase intracellular C_i_ pools ~6-fold higher than that permissible by passive diffusion (Fig. 2B (Burns and Beardall, 1987; Colman and Rotatore, 1995; Badger *et al.*, 1998).

The δ isoform of CA (or TWCA1), whose expression in the diatom *T. weissflogii* is modulated by extracellular CO_2_ levels (Morel *et al.*, 1994; Lane and Morel, 2000a, b) and in marine dinoflagellates functions as an external CA (Lapointe *et al.*, 2008), holds the potential as a key component of a CCM in microalgae. The catalytic activity and inhibition of δ-CA demonstrate the functionality of the CA in the diatom *Thalassiosira pseudonana* and the haptophyte *Emiliania huxleyi* (Soto *et al.*, 2006; Lee *et al.*, 2013; Del Prete *et al.*, 2014). Using sequence homology searches, we were able to detect δ-CA homologs in genome data sets for *P. carterae* and *T. lutea*, but not in *P. tricornutum* or *P. lutheri* (using the Pavlovale sp. CCMP2436 genome as a proxy). Importantly the absence of detectable sequence homology does not disqualify these microalgae from producing δ-CA or alternative CA isoforms, especially considering that new CAs are still being discovered (Jin *et al.*, 2016; Kitkanti *et al*., 2016). Indeed, a number of other CA types are expressed in *P. tricornutum* that include one localized in the pyrenoid (Tachibana *et al.*, 2011), an extracellular CA (Hopkinson *et al.*, 2013), and a θ-type CA located in the thylakoid lumen (Jin *et al.*, 2016). Our BLAST search of the Pavlovale sp. CCMP2436 genome supports the absence of a δ-CA in *P. lutheri*; however, further investigation is required (e.g. whether *P. lutheri* contains other forms of CA). Overall, the existing evidence suggests that the presence of a pyrenoid coincides with the presence and activity of CA (Fig. 2B), consistent with their role in the microalgae CCM.

### The carboxylation properties of haptophyte Rubisco

**Fig. 3.**
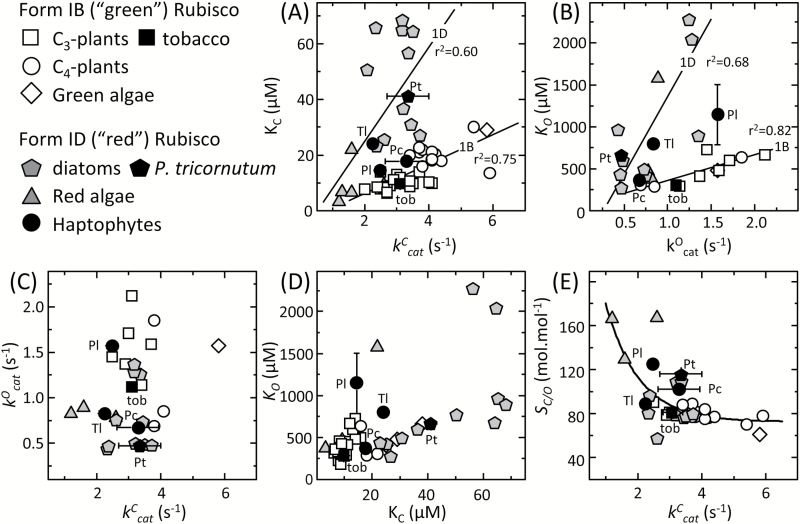
The diversity in the kinetic properties of haptophyte Rubisco at 25 °C. Comparative relationships between the kinetic properties measured in this study for Rubisco from *P. lutheri* (Pl), *P. carterae* (Pc), *T. lutea* (Tl), the diatom *P. tricornutum* (Pt), and from tobacco (Tob) with those of other Form 1B and 1D Rubiscos (see key) as curated by Young *et al.* (2016). The plotted maximal carboxylation and oxygenation turnover rates (*k*^C^_cat_, *k*^O^_cat_), relative specificity for CO_2_ over O_2_ (*S*_C/O_). and the Michaelis constants (*K*_m_) for CO_2_ and O_2_ (*K*_C_, *K*_O_) are from Table 1. Linear regressions are shown for the differing (A) *k*^C^_cat_–*K*_C_ and (B) *k*^O^_cat_–*K*_*O*_ relationships displayed for Form 1B and 1D Rubiscos. No statistically significant relationships were evident among correlative analyses of (C) *k*^C^_cat_ and *k*^O^_cat_*K*_C_ or between (D) *K*_C_ and *K*_O_. (E) An exponential relationship was apparent when comparing the kinetic trade-off between *S*_C/O_ with *k*^***^C^***^_***_cat_***_ with the differing phylogenetic Rubisco groupings aggregated at differing positions along the gradient

Form 1B Rubisco from organisms operating a CCM characteristically show higher rates of maximum carboxylation *k*^C^_cat_ and a reduction in CO_2_ affinity (i.e. an increase in *K*_C_) than the Rubisco from their non-CCM relatives. For example, the Rubisco from C_4_ plants typically have a higher *k*^C^_cat_ and higher *K*_C_ than C_3_ plant Rubisco (Sage, 2002; Savir *et al.*, 2010; Sharwood *et al.*, 2016a, c; Tcherkez, 2016). As shown in Fig. 3A, the *K*_C_ diversity among Form 1B vascular plant Rubisco spans a limited range in values relative to the Form 1D Rubisco from diatoms (Hanson, 2016; Young *et al.*, 2016). Moreover, the relationship between *K*_C_ and *k*^C^_cat_ at 25 °C for the Form 1D Rubisco differs from Form 1B Rubisco (Fig. 3A).

For comparison of different CCM effectiveness on Rubisco kinetics within organisms containing the 1D Rubisco, we examined the kinetics of the Form 1D Rubiscos from freshly lysed *P. carterae*, *T. lutea*, and *P. lutheri* cells. The Rubisco activity in the cellular extract was stable at 25 °C for at least 20 min following extraction (see Supplementary Fig. S1 at *JXB* online). The CO_2_–Mg^2+^ activation status of the extracted Rubisco varied between 50% and 60%, comparable with that seen in the cellular extract of diatoms (Young *et al.*, 2016). To ensure full activation of all eight catalytic sites in each L_8_S_8_ molecule, the cellular extract was incubated for 10–15 min at 25 °C in buffer containing 10 mM MgCl_2_ and 10 mM NaHCO_3_ before assaying *k*^C^_cat_ under varying CO_2_ concentrations by ^14^CO_2_ fixation. By this approach, the values of *k*^C^_cat_ and *K*_C_ extrapolated from fitting the data to the Michaelis–Menten equation were reproducible between replicate cellular preparations (Table 1).

**Table 1.** Rubisco kinetic parameters measured at 25 °C

Species	*k* ^C^_cat_ (s^–1^)	*K*_C_ (µM)	*K*_O_ (µM)	*S*_c/o_ (mol mol^–1^)	*K*_C_^21% O2^ (µM)	*k* ^O^_cat_ (s^–1^)	*k* ^O^_cat_ */K*_O_ (mM^–1^ s^–1^)	CE	
								*k* ^ C^_ cat_/*K*_*C*_ (mM^ –1 ^ s ^ –1 ^)	*k* ^ C ^_ cat _ *K*_ C _^ 21%O2 ^ (mM ^ –1 ^ s ^ –1 ^)
**Haptophytes**									
*P. carterae*	3.3 ± 0.4	17.7 ± 1.5	366 ± 60	102 ± 1	30.6	0.7	1.8	186	108
*T. lutea*	2.2 ± 0.1	24.1 ± 0.5	800 ± 55	89 ± 1	32.2	0.8	1.0	91	68
*P. lutheri*	2.5 ± 0.1	14.5 ± 1.6	1146 ± 212	125 ± 2	17.8	1.6	1.4	172	140
**Diatom** ^***a***^									
*P. tricornutum*	3.3 ± 0.5	41.1 ± 1.3	664 ± 54	116 ± 2	57.6	0.5	0.7	80	55
**C**_**3**_ **plant** ^***a***^									
*N. tabacum*	3.1 ± 0.3	9.7 ± 0.1	283 ± 15	81 ± 1	18.9	1.1	3.9	319	164

CE, carboxylation efficiency.

The rate of oxygenation (*k*^O^_cat_) was calculated using the equation *k*^O^_cat_=(*k*^C^_cat_×*K*_O_)/(*K*_C_×*S*_C/O_)×*K*_C_ at 25 °C under ambient atmospheric O_2_ levels; *K*_C_^21%O2^ was calculated as *K*_C_(1+[O_2_]/*K*_O_) assuming an O_2_ solubility of 0.00126 mol (l bar)^–1^ and an atmospheric pressure of 1.013 bar resulting in an [O_2_] value of 267 µM in solution.

Values shown are average of measurements from *n*≥3 (±SD) biological repeat samples.

^*a*^Data from Young *et al.* (2016).

Significant kinetic diversity at 25 °C was observed among each haptophyte Rubisco relative to *P. tricornutum* (model diatom species) and *Nicotiana tabacum* (tobacco, model plant Rubisco used in kinetic comparisons; Whitney *et al.*, 2001; Sharwood *et al.*, 2016a, b) (Table 1). The *k*^C^_cat_ of *P. carterae*, *P. tricornutum*, and tobacco were similar and each ~50% higher than those of *T. lutea* and *P. lutheri* Rubisco. Comparable levels of variation in *K*_C_ were also observed among the haptophyte Rubiscos (14.5–24.1 μM) that are notably lower and spanning a smaller range than the *K*_C_ values of diatom Rubiscos (22–70 μM; Fig. 3A). This suggests haptophyte Rubisco may experience a lower CO_2_ microenvironment relative to diatoms. This is probably the case for Rubisco in *P. lutheri* that lacks a pyrenoid (Burris, 1981; Bendif *et al.*, 2011) and whose Rubisco has the lowest *K*_C_ and highest *S*_C/O_ (i.e. a greater selectivity for CO_2_ over O_2_; Table 1).

### A correlative analysis of haptophyte Rubisco kinetics

A comparison of Rubisco kinetics of each haptophyte identified contrasting relationships when compared with the 25 °C properties of Form 1B and 1D L_8_S_8_ Rubisco from a range of eukaryotic phototrophs (Fig. 3A–E; data compiled in Supplementary Table S1). An examination of the *K*_C_–*k*^C^_cat_ relationship for ‘green’ Form 1B (vascular plants, CCM-positive green algae) and ‘non-green’ Form 1D (diatoms, haptophytes, and red algae) Rubisco suggests they follow differing trajectories (Fig. 3A). As suggested previously, this might arise from lineage-dependent variation in ribulose bisphosphate (RuBP) enolization energies and/or mechanistic differences in their multistep carboxylation chemistry (Tcherkez, 2013, 2016; Young *et al.*, 2016).

With regard to haptophyte Rubisco, the *K*_C_–*k*^C^_cat_ relationship of *P. lutheri* and *P. carterae* Rubisco appeared to align more closely with Form 1B Rubisco (Fig. 3A). Consequently, their carboxylation efficiencies under both anaerobic (*k*^C^_cat_/*K*_C_) and ambient O_2_ (*k*^C^_cat_/*K*_C_^21%O2^) are higher than those of *T. lutea* Rubisco (Table 1) whose *K*_C_–*k*^C^_cat_ relationship closely aligns with red algae and diatom Form 1D Rubiscos (Fig. 3A), and lower carboxylation efficiency of *P. tricornutum* Rubisco (Table 1). These findings suggest that there may be differences in the CCM effectiveness between the bulging pyrenoids in *P. carterae* relative to the immersed pyrenoids of diatoms and *T. lutea.* It also raises questions regarding how differences in the CCM and/or cellular metabolism in phytoplankton with immersed pyrenoids have led to the evolution of atypical Form 1D Rubisco kinetics.

Somewhat analogous to their differing *K*_C_–*k*^C^_cat_ relationships, both Form 1B and 1D enzymes showed differing linear correlations between *K*_O_ and *k*^O^_cat_ (Fig. 3B) but no identifiable correlations between their *k*^C^_cat_/*k*^O^_cat_ (Fig. 3C) and *K*_C_/*K*_O_ (Fig. 3D) relationships. This finding is consistent with increasing experimental evidence that changes in the carboxylation and oxygenation properties are not coupled in an obligatory manner (Savir *et al.*, 2010; Whitney *et al.*, 2011; Sharwood *et al.*, 2016*a*; Sharwood, 2017). This unfastening of carboxylation and oxygenation has enabled significant Rubisco kinetic diversity to have evolved in nature, in particular with regard to CO_2_/O_2_ selectivity (*S*_C/O_) whose correlative trade-off with *k*^C^_cat_ follows a diffuse exponential relationship (Fig. 3E; Sharwood, 2017) rather than the linear response previously postulated (Tcherkez *et al.*, 2006; Savir *et al.*, 2010). Within the *k*^C^_cat_–*S*_C/O_ relationship, the haptophyte Rubisco localizes in a region comparable with diatom Rubisco between the high *S*_C/O_, low *k*^C^_cat_ of Form 1D red algae Rubisco and the lower *S*_C/O_, higher *k*^C^_cat_ of Form 1B Rubisco (Fig. 3E).

### Interpreting the differing O_2_ sensitivities of haptophyte Rubisco

While the CCMs of phototrophic organisms function to elevate the CO_2_:O_2_ ratio around Rubisco through increased CO_2_ supply, it is unclear how the ratio is dependent on complementary mechanisms to lower O_2_. In many organisms employing a CCM, the O_2_-generating components are located away from Rubisco. For example, the Rubisco-containing bundle sheath cell (BSC) chloroplasts in C_4_ plants within NADP-malic enxyme (NADP-ME) subtypes characteristically lack the O_2_-evolving PSII complexes (Sage *et al.*, 2014; von Caemmerer and Furbank, 2016). Similarly, the thylakoids traversing the pyrenoid of the red algae *Porphyridium cruentum* lack PSII (McKay and Gibbs, 1990), while in the dinoflagellate *Gonyaulax polyedra* during times of high carbon fixation the Rubisco is spatially relocated to pyrenoids near the cell center away from the O_2_-evolving light-harvesting reactions (Nassoury *et al.*, 2001). Similarly in cyanobacteria, the carboxysomes localize to the cell interior away from the PSII thylakoids lining the cell periphery (Liberton *et al.*, 2011). These strategies for spatially separating O_2_ production away from Rubisco appear to be key components of the CCM. Unfortunately, measuring the O_2_ concentration in bundle sheath cell chloroplasts, cyanobacteria carboxysomes, or inside the pyrenoid of algae remains an insurmountable challenge.

**Fig. 4.**
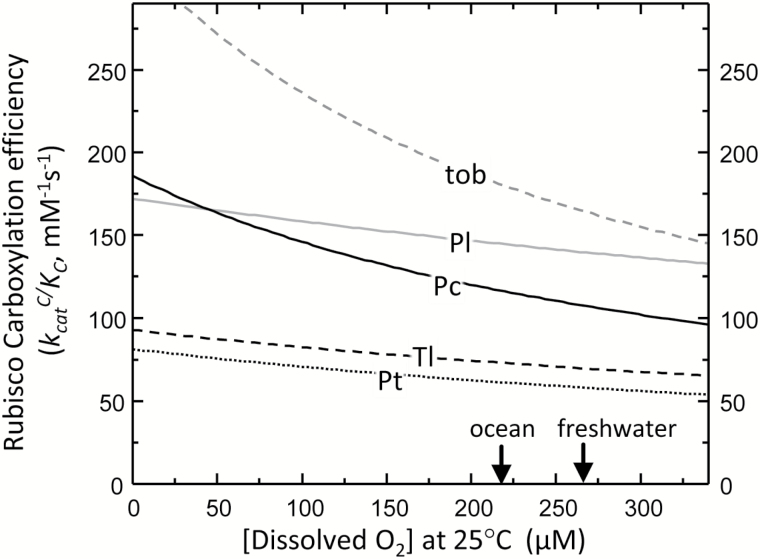
The differential effect of O_2_ on Rubisco carboxylation efficiency. Variation in the response of carboxylation efficiency (CE; *k*^C^_cat_/*K*_C_) to O_2_ levels (*O*) for Rubisco from tobacco (tob, vascular plant control), the diatom *P. tricornutum* (Pt, dotted line), and the haptophytes *P. lutheri* (Pl, solid gray line), *P. cartera*e (Pt, solid black line), and *T. lutea* (Tl, dashed black line). Lines were fitted to the equation CE=*k*^C^_cat_/{*K*_C_×[1+(*O*/*K*_O_)]} using the parameters listed in Table 1. Arrows indicate the differing O_2_ levels in fresh water and the ocean surface [assuming ~3.5% (w/v) salinity] at an atmospheric pressure of 1.013 bar.

As indicated above, the oxygenation properties of Rubisco show significant natural variation. Drawing correlations between their O_2_ sensitivity (i.e. *K*_O_) and CCM efficiency is therefore quite challenging. An additional complexity is that the extent of O_2_ solubility is reduced by the highly proteinaceous matrix of pyrenoids, chloroplasts, and carboxysomes (Tcherkez, 2016) and by the increasing pressures experienced by microalgae down the water column. Interestingly the higher *K*_O_ of *T. lutea*, *P. tricornutum*, and *P. lutheri* Rubisco lend to limiting the influence of changing O_2_ concentrations on carboxylation efficiency relative to Rubisco from tobacco and *P. carterae* (Fig. 4). This implies that the variable O_2_ concentrations experienced by diatoms and haptophytes within the water column might have influenced their Rubisco kinetic evolution. Furthermore, pyrenoid morphology and ultrastructure may also have influenced the evolved oxygenase properties. For example, *P. lutheri* Rubisco exhibits a low affinity for O_2_ (*K*_O_ ~1150 μM), implying that its Rubisco experiences higher O_2_ concentrations than the Rubisco from algae possessing immersed (e.g. *T. lutea*, *P. tricornutum*, *K*_O_ ~650–800 μM) or bulging (*P. carterae*, *K*_O_ ~366 μM) pyrenoids (Table 1).

These observations could be interpreted to suggest that the pyrenoids, in particular those with a bulging morphology, might be lowering the O_2_ environment to augment the CO_2_:O_2_ ratio around Rubisco. Possibly internally bulging pyrenoids may locate Rubisco closer to the reducing chemistry of the cytosol or the mitochondria and their respired CO_2_. Other mechanisms for altering pyrenoid CO_2_:O_2_ include reducing thylakoid number within the pyrenoid, reducing the O_2_-producing PSII activity (McKay and Gibbs, 1990), or employing pyrenoid tubules for diffusion (Engel *et al.*, 2015). Challenging these hypotheses is the observed high K_O_ (~2000 μM, indicating an insensitivity to O_2_) for Rubisco from the pyrenoid-containing diatom *Thalassiosira weissflogii* (Young *et al.*, 2016) and that multiple thylakoids traverse the pyrenoid of *P. carterae*, albeit with untested levels of PSII activity (Manton and Peterfi, 1969).

### The influence of a CCM on the Rubisco requirement in haptophytes

A characteristic feature of the CCM in C_4_ plants is that it reduces the requirement for Rubisco, allowing for increased nitrogen use efficiency (Ghannoum *et al.*, 2005). In diatoms, the Rubisco content was found to correlate with *K*_C_, suggesting that the allocation of resources into the enzyme may depend on CCM efficiency (Young *et al.*, 2016). For example, the low *K*_C_ of Rubisco from *Phaeodactylum* and *Chaetoceros* diatom species correlated with increased investment in Rubisco content, while in *Thalassiosira* and *Skeletonema* species it was hypothesized that resources were instead allocated to the CCM rather than Rubisco to saturate the enzymes low CO_2_ affinity (Young *et al.*, 2016).

**Fig. 5.**
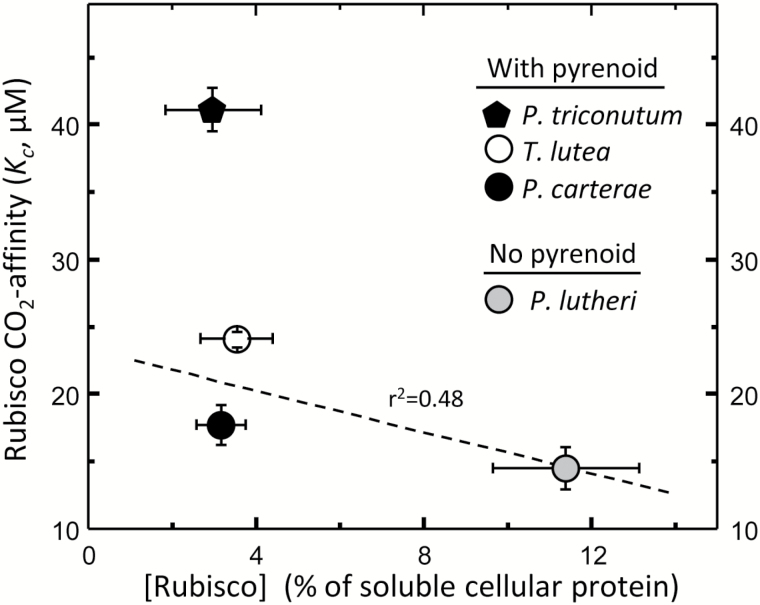
Rubisco content is reduced in pyrenoid-containing phytoplankton. The Rubisco content (quantified by [^14^C]CABP binding and expressed as a percentage of the cellular soluble protein) in cells grown at 20 °C under saturating nutrients was higher (11.4 ± 1.2%) in the pyrenoid-lacking *P. lutheri* cells relative to that in *P. cartera*e (3.0 ± 0.8%), *T. lutea* (3.5 ± 0.9%), and *P. tricornutum* (3.2 ± 0.6%).

Among the three haptophyte species examined here, we identified a negative relationship between increasing CO_2_ affinity (i.e. reducing *K*_C_) and increasing Rubisco content (dashed line, Fig. 5). The trajectory of this relationship poorly correlated with the Rubisco content and *K*_C_ of *P. tricornutum*. While it is known that Rubisco content in diatoms can be influenced by growth stage (Losh *et al.*, 2013), our measurements comprised replicate algae samples from cultures growing under non-nutrient limiting conditions and resulted in reproducible measures of Rubisco content (Fig. 5). While future experiments are aimed at examining these properties from a wider range of microalgae species, it is apparent that in the pyrenoid-lacking *P. lutheri* cells there is ~3- to 4-fold higher investment of soluble cellular protein in Rubisco (Fig. 5). Likewise, the Rubisco content in hornworts also shows a comparable correlation with the presence/absence of pyrenoids (Hanson *et al.*, 2002). Similarly, in C_3_ plants, Rubisco comprises a larger resource investment [25–50% (w/w) total soluble protein] relative to C_4_ plants where the CCM and higher *k*^C^_cat_ reduces the amount of Rubisco required [i.e. 8–15% (w/w) of the soluble cellular protein (Ghannoum *et al.*, 2005; Sharwood *et al.*, 2016a, c)].

### How suited is phytoplankton Rubisco to supporting photosynthesis in C_3_ plants?

**Fig. 6.**
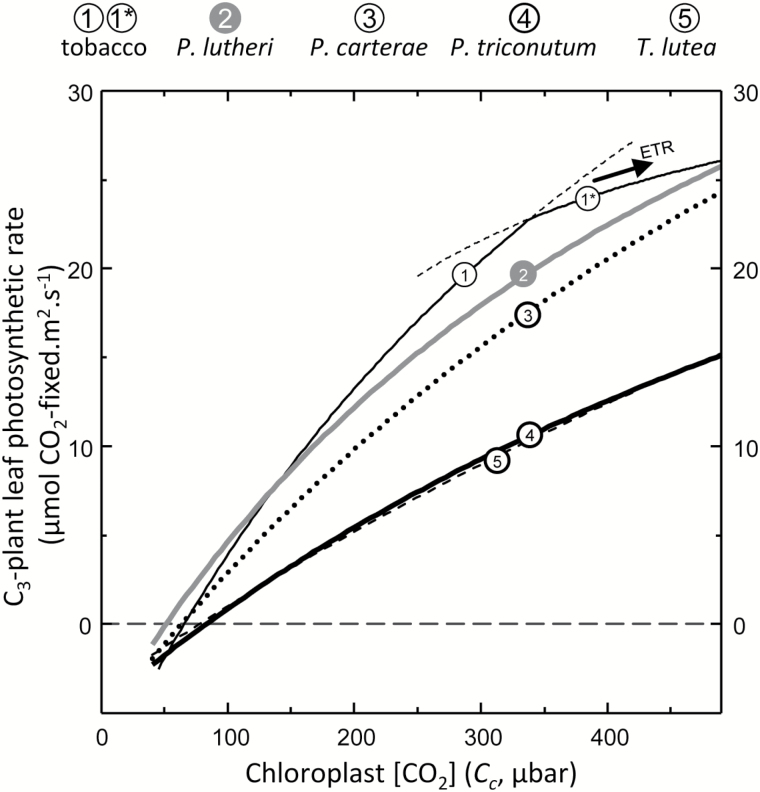
The varying potential of phytoplankton Rubisco in a C_3_ leaf. The influence of each Rubisco analyzed in Table 1 on CO_2_ assimilation rates (*A*) at 25 °C in a C_3_ leaf as a function of *C*_c_ was modeled according to Farquhar *et al.* (1980) as described in the Materials and methods. For the tobacco Rubisco, the photosynthetic rate became light limited (indicated by 1*) at *C*_*c*_>320 μbar.

Improving the catalytic efficiency of Rubisco is a key target for improving the rate of photosynthesis and growth in key C_3_ crops such as rice and wheat (Long *et al.*, 2015; Sharwood *et al.*, 2016*b*). This has led to considerable interest in identifying whether the natural catalytic diversity of Rubisco can be exploited to deliver improvements in crop Rubisco performance. The faster Rubisco from *Synechococcus* PCC7942 (cyanobacteria) and the photosynthetic bacterium *Rhodospirillum rubrum* are not able to support faster C_3_ plant growth, even under elevated CO_2_, due to their low carboxylation efficiencies under ambient O_2_ (*k*^C^_cat_/*K*_C_^21%O2^) and their low *S*_C/O_ (Sharwood, 2017). In comparison, the higher *k*^C^_cat_/*K*_C_^21%O2^ and *S*_C/O_ of the Form 1D Rubisco from *Griffithsia monilis* (filamentous red algae) would support faster rates of photosynthesis in C_3_ crops with the potential to improve productivity by up to 30% (Long *et al.*, 2015). Realizing this benefit is impeded by the incompatible assembly requirements of Form 1D Rubisco in plant chloroplasts (Whitney *et al.*, 2001). Nevertheless, it is hoped solutions to improving crop Rubisco may be achieved through increased understanding of natural kinetic diversity among all Rubisco forms (Whitney *et al.*, 2011; Sharwood, 2017). We therefore used the modeling approach of Farquhar *et al.* (1980) to simulate how each microalga would influence C_3_ photosyntesis under varying chloroplast CO_2_ concentrations (*C*_c_; Fig. 6). The low *k*^C^_cat_/*K*_C_^21%O2^ of *T. lutea* (and *P. tricornutum*) Rubisco (Table 1) impeded their simulated effect on C_3_ photosythesis at 25 °C. In contrast, the high *S*_C/O_ of *P. lutheri* Rubisco improved the simulated photosynthetic rates relative to tobacco Rubisco under low *C*_c_ but not above 150 μbar of CO_2_ due to its lower *k*^C^_cat_ and *k*^C^_cat_/*K*_C_^21%O2^ (Table 1). This finding suggests hat future kinetic surveys to identify microalgae Rubisco better suited to operating in C_3_ plant chloroplasts might best focus on microalgae that lack a pyrenoid and a CCM.

### Conclusions and future directions

Due to their importance at the base of the marine food chain, global biogeochemical cycling, and interpretation of paleo-chemical signals in marine sediments, it is essential that we better understand the diversity and function of the algae CCMs. In this pilot study, we provide preliminary evidence for correlations between Rubisco content, kinetics, and pyrenoid morphology within the chloroplast of different haptophytes and the diatom *P. tricornutum*. Recent work has highlighted the lack of knowledge on the components and variable efficiency of the CCM across environmentally important microalgae (Hanson, 2016; Hopkinson *et al.*, 2016; Young *et al.*, 2016). Previous models of the algal CCM relied on correlations with distant photosynthetic organisms and limited Rubisco kinetic data. Elucidating the diversity and biological relevance of CCMs in these species will provide the groundwork necessary for understanding primary production in the world’s oceans.

Our study provides evidence for the potential to use the binding affinities of Rubisco as a probe to gauge the intracellular CO_2_:O_2_ ratio around Rubisco and which might include an oxygen exclusion function by the pyrenoid. Future correlative analyses of Form 1D and Form 1B Rubisco kinetics from microalgae lacking pyrenoids and with differing pyrenoid morphologies are needed to yield a more robust functional understanding of the intrapyrenoid microenvironment and the natural diversity in carbon fixation in both terrestrial and aquatic ecosystems. Although challenging to measure, these Rubisco analyses are essential for understanding both (i) the different evolutionary histories of Form I Rubisco whose kinetics appear to have divergently evolved and (ii) the extent to which the competing carboxylation and oxygenation properties can be decoupled. Refining the existing assumptions about diversity and trends in photosynthetic evolution are paramount. Included in such endeavors are whether the non-canonical correlation between *K*_C_ and *k*^C^_cat_ is limited to Form 1D Rubisco from microalgae with immersed pyrenoids (Fig. 3A), if resource allocation to Rubisco is elevated in species with a less effective CCM (Fig. 5), and to what extent increases in *k*^C^_cat_/*K*_C_^21%O2^, *S*_C/O_, and *K*_O_ (i.e. a reduced O_2_ sensitivity) can be used as a proxy to gauge the effectiveness of microalgae CCMs.

## Supplementary data

Supplementary data are available at *JXB* online.

Fig. S1. Measurement of Rubisco activation status and stability *in vitro* at 25 °C.

Table S1. Rubisco kinetics at 25 °C as shown in Fig. 3. 

## Supplementary Material

supplementary_figure_S1

supplementary_table_S1
