# Supplementary material for: The role of Rubisco kinetics and pyrenoid morphology in shaping the CCM of haptophyte microalgae
Source: J Exp Bot. 2017 Jun 3;68(14):3959–69. doi: 10.1093/jxb/erx179 (PMC5853415; doi:10.1093/jxb/erx179)
Supplement: supplementary_figure_S1 [file erx179_suppl_supplementary_figure_s1.pdf]

Supplemental data

The role of Rubisco kinetics and pyrenoid morphology in shaping the CCM of Haptophyte microalgae

Ana M. C. Heureux, Jodi N. Young, Spencer M. Whitney, Maeve R. Eason-Hubbard, Renee B. Y. Lee, Robert E. Sharwood, Rosalind E. M. Rickaby

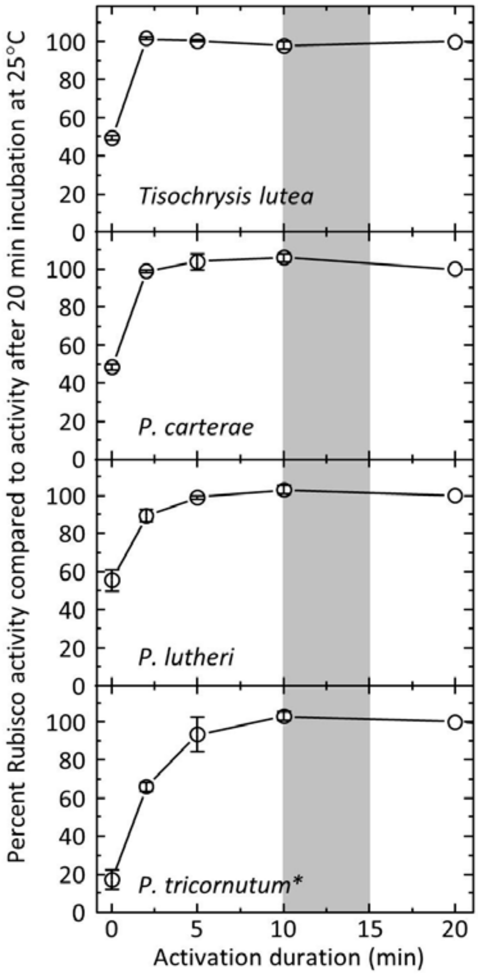

Figure S1. Measurement of Rubisco activation status and stability *in vitro* at 25°C.

Soluble cellular protein from the phytoplankton species indicted was rapidly extracted in CO_2-free extraction buffer (containing 5 mM $MgCl_2$) and used to measure changes in Rubisco $^{14}CO_2$-fixation rate after activating the extract for 0 to 20 min in buffer containing 15 mM $MgCl_2$ and 15 mM $NaHCO_3$ at 25°C. Details of the carboxylase assay are described in (Young *et al.*, 2016). Grey shading indicates the time when protein extract was assayed to quantify k^C_{cat}, K_C and K_O (see Table 1 in main text). Data represents measures from duplicate biological samples (± SD). *, data from (Young *et al.*, 2016).

References

Young JN, Heureux AMC, Sharwood RE, Rickaby REM, Morel FMM, Whitney SM. 2016. Large variation in the Rubisco kinetics of diatoms reveals diversity among their carbon-concentrating mechanisms. Journal of Experimental Botany **67**, 3445-3456.
