# Supplementary material for: The role of Rubisco kinetics and pyrenoid morphology in shaping the CCM of haptophyte microalgae
Source: J Exp Bot. 2017 Jun 3;68(14):3959–69. doi: 10.1093/jxb/erx179 (PMC5853415; doi:10.1093/jxb/erx179)
Supplement: supplementary_table_S1 [file erx179_suppl_supplementary_table_s1.pdf]

Table S1: Rubisco kinetics at 25°C as shown in Figure 3

	k_{cat}^{c} (s^{-1})	K_C (µM)	K_O (µM)	Sc/o ($mol.mol^{-1}$)	calc k_{cat}^{o} (s^{-1})	Dataset reference
**Red Algae**						
Griffithsia monilis	2.6	9.3	467	167	0.8	Savir et. al. (2010)
Galdiera sulfuraria	1.2	3.3	374	166	0.8	Savir et. al. (2010)
Porphyridium cruentum	1.6	22	1574	129	0.9	Badger et. al. (1998)
Cyanidium caldarium	1.3	6.7	n.d.	n.i.	n.d.	Badger et. al. (1998)
Cyanidium partita	1.6	6.6	n.d.	n.i.	n.d.	Badger et. al. (1998)
**Green algae**						
Scenedesmus obliquus	n.d.	38	660	63	n.d.	Savir et. al. (2010)
Chlamydomonas reinhardtii	5.8	29	480	61	1.57	Savir et. al. (2010)
Euglena gracilis	n.d.	25	410	54	n.d.	Savir et. al. (2010)
**Gymnosperms**						
Basal angiosperm						
**C_3**						
Arabidopsis thaliana	4.1	10	333	n.d.	n.d.	Galmes et. al. (2014)
Crithmum maritimum	3.4	9	183	n.d.	n.d.	Galmes et. al. (2014)
Dactylis glomerata cv. Porto	3.2	11	453	n.d.	n.d.	Galmes et. al. (2014)
Eucalyptus moorei	3.2	10	285	n.d.	n.d.	Galmes et. al. (2014)
Eucalyptus neglecta	2.5	8	230	n.d.	n.d.	Galmes et. al. (2014)
Flaveria pringlei	3.1	12	666	81	2.1	Savir et. al. (2010)
Glycine max	n.d.	9	430	82	n.d.	Savir et. al. (2010)
Iris douglasiana	3.5	10	413	n.d.	n.d.	Galmes et. al. (2014)
Limonium latebracteatum	2.7	9	344	n.d.	n.d.	Galmes et. al. (2014)
Limonium stenophyllum	2.6	8	457	n.d.	n.d.	Galmes et. al. (2014)
Limonium virgatum	2.4	9	381	n.d.	n.d.	Galmes et. al. (2014)
lolium perenne	n.d.	16	500	80	n.d.	Galmes et. al. (2014)
Nicotiana tabacum	3.1	10	283	83	1.1	Young et. al. (2016)
Pallenis maritima	2.7	6	321	n.d.	n.d.	Galmes et. al. (2014)
Sideritis cretica subsp. spicata	2.0	8	328	n.d.	n.d.	Galmes et. al. (2014)
Spnacia oleracea	3.7	14	480	80	1.59	Savir et. al. (2010)
Tetragonium expnasa	n.d.	13	600	81	n.d.	Savir et. al. (2010)
Teucrium heterophyllum	2.7	7	359	n.d.	n.d.	Galmes et. al. (2014)
Trachycarpus fortunei	2.8	9	364	n.d.	n.d.	Galmes et. al. (2014)
Triticum aestivum	2.5	14	730	90	1.5	Savir et. al. (2010)
Triticum aestivum cv. Alexandria	4.0	10	414	n.d.	n.d.	Galmes et. al. (2014)
**C_4**						
Amaranthus edulis	4.1	18	289	77	0.9	Whitney et. al. (2011), Savir et. al. (2010)
Amaranthus hybridus	3.8	16	640	82	1.9	Whitney et. al. (2011), Savir et. al. (2010)
Cynodon dactylon	3.7	21	n.d.	89	n.d.	Whitney et al (2011)
Flaveria australasica	3.8	22	309	77	0.7	Whitney et. al. (2011), Savir et. al. (2010)
Flaveria bidentis	4.2	20	n.d.	76	n.d.	Whitney et. al. (2011)
Flaveria kochiana	3.7	23	n.d.	77	n.d.	Whitney et. al. (2011)
Flaveria trinervia	4.4	18	n.d.	77	n.d.	Whitney et. al. (2011)
Paspalum dilatatum	3.4	20	n.d.	88	n.d.	Whitney et. al. (2011)
Sorghum bicolor	5.4	30	n.d.	70	n.d.	Whitney et. al. (2011)
Zea mays	5.5	19	397	88	1.3	Young et. al. (2016)
Zoysia japonica	4.1	19	n.d.	84	n.d.	Whitney et. al. (2011)
**Diatoms**						
Thalassiosira weissflogii (CCMP 1336)	3.2	65	2032	79	1.3	Young et. al. (2016)
Thalassiosira oceania (CS-427)	2.4	65	954	80	0.4	Young et. al. (2016)
Skeletonema marinoi (CCMP 1332)	3.2	68	883	n.d.	n.d.	Young et. al. (2016)
Chaetoceros calcitrans (CCMP 1315)	2.6	25	413	57	0.8	Young et. al. (2016)
Chaetoceros muelleri (CCMP 1316)	2.4	23	425	96	0.5	Young et. al. (2016)
Chaetoceros calcitrans (CS-178)	3.4	31	490	75	0.7	Young et. al. (2016)
Bellerochea cf. horologicalis (CS-874/01)	2.1	50	764	n.d.	n.d.	Young et. al. (2016)
Phaeodactylum tricornutum (UTEX 642)	3.2	36	592	108	0.5	Young et. al. (2016)
*Phaeodactylum tricornutum (*CS-29)	3.3	41	664	116	0.5	this study
Fragilariopsis cylindrus (CCMP 1102)	3.5	64	667	77	0.5	Young et. al. (2016)
Cylindrotheca fusiformis (CS-13)	3.7	n.d.	n.d.	79	n.d.	Young et. al. (2016)

**Haptophytes**						
Pleurochrysis carterae (CS-287)	3.3	18	366	102	0.7	this study
Tisochrysis lutea (CS-177)	2.2	24	800	89	0.8	this study
Pavlova lutheri (CS-182)	2.5	15	1146	125	1.6	this study

n.d. = not determined

n.i. = not included

References

Badger MRT, Andrews J, Whitney SM, Ludwig M, Yellowlees DC, Leggat W, Price GD (1998) The diversity and coevolution of Rubisco, plastids, pyrenoids, and chloroplast-based CO_2-concentrating mechanisms in algae. Canadian Journal of Botany **76**: 1052-1071

Galmes J, Kapralov MV, Andralojc PJ, Conesa MÀ, Keys AJ, Parry MAJ, Flexas J (2014) Expanding knowledge of the Rubisco kinetics variability in plant species: environmental and evolutionary trends. Plant, Cell & Environment **37:** 1989-2001

Savir Y, Noor E, Milo R, Tlusty T (2010) Cross-species analysis traces adaptation of Rubisco toward optimality in a low-dimensional landscape. Proceedings of the National Academy of Sciences **107**: 3475-3480

Whitney SM, Houtz RL, Alonso H (2011) Advancing Our Understanding and Capacity to Engineer Nature's CO_2-Sequestering Enzyme, Rubisco. Plant Physiology **155**: 27-35

Young, J. N., Heureux, A. M., Sharwood, R. E., Rickaby, R. E., Morel, F. M., & Whitney, S. M. (2016). Large variation in the Rubisco kinetics of diatoms reveals diversity among their carbon-concentrating mechanisms. Journal of Experimental Botany, **67**(11): 3445-3456.
